# Seroprevalence of poliovirus antibodies in Nigeria: refining strategies to sustain the eradication effort

**DOI:** 10.11604/pamj.supp.2023.45.2.38098

**Published:** 2023-07-13

**Authors:** Omotayo Bolu, Usman Adamu, Richard Franka, Chukwuma David Umeokonkwo, Qian An, Stacie Greby, Sharla McDonald, Bernardo Mainou, Nwando Mba, Ndidi Agala, Wiedad Roodly Archer, Fiona Braka, Sume Gerald Etapelong, Tegegne Sisay Gashu, Anisur Rahman Siddique, Adeyelu Asekun, McPaul Okoye, Nnaemeka Iriemenam, Eric Wiesen, Mahesh Swaminathan, Chikwe Ihekweazu, Faisal Shuaib

**Affiliations:** 1 Centers for Disease Control and Prevention, Abuja, Nigeria,; 2 National Primary Health Care Development Agency, Abuja, Nigeria,; 3 Africa Field Epidemiology Network, Abuja, Nigeria,; 4 Nigeria Center for Disease Control, Abuja, Nigeria,; 5 Institute of Human Virology, Abuja, Nigeria; 6 World Health Organization, Abuja Country Office, Abuja, Nigeria,; 7 United Nations Children Fund, Abuja, Nigeria

**Keywords:** Prevalence, poliomyelitis, antibodies, immunization, risk factors, polio virus vaccine, immunity, disease outbreaks, serotype, Nigeria

## Abstract

**Introduction:**

in 2016, a switch from trivalent oral poliovirus vaccine (OPV) (containing serotypes 1,2,3) to bivalent OPV (types 1,3) was implemented globally. We assessed the seroprevalence of poliovirus antibody levels in selected Nigerian states, before and after the switch, documented poliovirus type2 outbreak responses conducted and ascertained factors associated with immunity gaps based on seroprevalence rates.

**Methods:**

we conducted a secondary analysis of stored serum samples from the 2018 Nigeria National HIV/AIDS Indicator and Impact Survey. Serum from 1,185 children aged 0-119 months residing in one southern and four northern states were tested for serotype-specific PV neutralizing antibodies; seropositivity was a reciprocal titer ≥8. We conducted regression analysis to determine sociodemographic risk factors associated with low seroprevalence using SAS 9.4.

**Results:**

children aged 24-119 months (pre-switch cohort) had seroprevalence against PV1, PV2, and PV3, of 97.3% (95% CI:96.4-98.2), 93.8% (95% CI:92.2-95.5), and 91.3% (95% CI:89.2-93.4), while children aged <24 months (post-switch) had seroprevalence of 86.0% (95% CI:81.2-90.8), 55.6% (95% CI: 47.7-63.4), and 77.2% (95% CI:71.0-83.4) respectively. Regression analysis showed age <24 months was associated with lower seroprevalence against all PV serotypes, (p<0.0001); females had lower seroprevalence against PV1 (p=0.0184) and PV2 (p=0.0354); northern states lower seroprevalence against PV1 (p=0.0039), while well-water source lower seroprevalence against PV3 (p=0.0288).

**Conclusion:**

this study showed high seroprevalence rates against PV 1, 2, and 3 in pre-switch children (aged 24-119 months). However, post-switch children (<24 months) had low immunity against PV2 despite outbreak responses. Strategies to increase routine immunization coverage and high-quality polio campaigns can increase immunity against polio virus.

## Introduction

During the 41st World Health Assembly in 1988, World Health Organization (WHO) Member States committed to the global eradication of poliomyelitis [1]. Oral poliovirus vaccine (OPV) is a highly effective public health intervention deployed to achieve polio eradication and has reduced the burden of polio by more than 99.99% globally since 1988. Wild poliovirus (WPV) type 2 and WPV type 3 were certified eradicated globally in 2015 and 2019, respectively [2,3]. Afghanistan and Pakistan remain the only two countries endemic with WPV type 1 after the certification of countries of the WHO African Region, including Nigeria, as WPV-free in August 2020 [4].

The live, attenuated Sabin OPV used for vaccination, while undergoing prolonged transmission in under-immunized populations, can genetically revert to a virulent strain called vaccine-derived poliovirus (VDPV). Vaccine-derived polioviruses are biologically equivalent to WPVs and can cause paralytic poliomyelitis among under-immunized or nonimmunized people. When transmission of VDPVs is confirmed, it is deemed an outbreak and the virus is denoted as a circulating (cVDPV). To prevent outbreaks of cVDPV type 2 (cVDPV2) emerging in areas with low polio immunity, the Global Polio Eradication Initiative (GPEI) implemented the global withdrawal of OPV type 2 for routine vaccination following certification of WPV type 2 eradication.

In April 2016, as an important aspect of OPV type 2 withdrawal, GPEI launched the globally synchronized switch from trivalent OPV (tOPV, containing Sabin strain types 1, 2, and 3) to the bivalent OPV (bOPV, containing only Sabin strain types 1 and 3). One dose of injectable inactivated poliovirus vaccine (IPV, containing types 1, 2 and 3) was introduced into routine immunization (RI) schedules to maintain some level of poliovirus immunity among children against all 3 poliovirus types [5] and reduce the risk of cVDPV2 paralytic disease.

After the African Region achieved WPV-free certification, the Region has continued to battle numerous, and some long-standing, outbreaks of paralytic poliomyelitis caused by cVDPV2 [6]. During 2016-2018, Nigeria reported 34 confirmed paralytic cases (as well as 58 viral isolates from healthy children and 47 from sewage samples). The 2016-2017 Nigeria Immunization Coverage Survey/Multiple Indicator Cluster Survey (NICS/MICS) [7], reported OPV3 coverage in children 12-23 months in several states as follows: Borno: 41.5%; Jigawa: 6.7%; Yobe: 9.4%; Sokoto: 7.1%; Lagos: 74.7%. Inactivated poliovirus vaccine was introduced in 2015, but implementation was staggered and varied by state, thus no IPV coverage was collected or reported at that time of the NICS/MICS 2016. However, the 2018 Demographic Health Survey (DHS, 2018) [8] for OPV3 and IPV coverage in children 12-23 months were as follows, respectively: Borno: 41%, 45%; Yobe: 43%, 40.4%; Jigawa: 48%, 36.5%; Sokoto: 12%, 20.8% and Lagos: 67%, 87%. The Nigeria Polio Eradication Program conducted eleven campaigns using monovalent Sabin OPV type 2 vaccine (mOPV2) to prevent and respond to cVDPV2 outbreaks between May 2016 and December 2018. Since the switch to bOPV, RI intensification at subnational levels using IPV was also strategically implemented in states with a high risk of cVDVP2 outbreaks.

The level of immunity against polio types 1, 2, and 3 from seroprevalence surveys in different states in Nigeria and time points before the interruption of WPV transmission revealed significant variability in population immunity [9-12]. We determined the seroprevalence of neutralizing poliovirus antibody levels in stored serum specimens collected from children during a nationwide 2018 Nigeria HIV serosurvey, following the withdrawal of tOPV and with the implementation of supplementary immunization activities (SIAs) with mOPV2 in cVDPV2 outbreak responses and assessed the factors associated with low seroprevalence. Findings on immunity gaps from this study will inform the development of evidence-based immunization strategies for outbreak responses and routine immunization intensification.

## Methods

**Study setting, design and population:** this was a secondary analysis of stored samples of the 2018 Nigeria National HIV/AIDS Indicator and Impact Survey (NAIIS). National HIV/AIDS Indicator and Impact Survey was a nationally representative, population-based, cross-sectional household survey conducted in all 36 states and the Federal Capital Territory (FCT) from July to December 2018 to determine national and subnational HIV incidence, prevalence, and viral load. Several variables were included in the data collection instruments; however, immunization histories were not collected. A total of 205,903 participants, including the caretakers of 32,354 children (aged 0-14 years) and 173,549 adults (aged 15-64 years), consented to storage and future testing of specimens for diseases of public health importance. This study and analysis focused on children aged 0 to 119 months (i.e. <10 years). The NAIIS used a two-stage cluster sampling technique, selecting enumeration areas followed by households and then participants. The details of sampling for the NAIIS survey have been reported [13]. Infants born after tOPV removal from the country´s immunization program in April 2016 to the time of sample collection of the NAIIS 2018 are defined as the post-switch population. Due to variability by state from the time of the tOPV switch to sample collection for NAIIS, post-switch children were restricted to children aged <24 months to reduce the risk of miscategorization of the post-switch population; pre-switch children were those aged 24-119 months included a fraction of post-switch children in the group aged 24-35 months.

**Blood samples collection and storage:** data and blood samples during NAIIS were collected from June-December 2018. Over 98% of participants in the NAIIS study granted consent for leftover samples to be stored and tested for other diseases of public health importance. Residual sera were stored at -80°C. Stored samples from four northern states (Borno, Jigawa, Yobo, Sokoto) and one southern state (Lagos) were selected as described below. Selected sera were shipped to the Population Immunity Laboratory at the Centers for Disease Control and Prevention in Atlanta, Georgia, USA. Samples were tested for the presence of neutralizing antibodies against poliovirus types 1, 2, and 3 using a standard microneutralization assay [14]. Seropositivity was defined as reciprocal titers of poliovirus neutralizing antibody ≥1:8.

**Sample size:** sample size for each state was determined based on the expected poliovirus seroprevalence using data from similar studies [9,10,12,15] and the target confidence interval (CI) adjusted for design effect. We assumed 60% overall seroprevalence in the northern states, 80% in Lagos State (a southern state), a target CI of 0.14, and a design effect of 2. The expected sample size was 1,224 for northern states (408 per state) with ~150 samples for children aged <24 months and 258 for 24-119 months per state; the sample size for Lagos State was 279. There were approximately 93 samples for each of the five age group strata. The total estimated sample size was 1,508. Due to the variability in poliovirus seroprevalence by age and the likelihood of lower seroprevalence among children aged <24 months, specimens for all children in this age group with consent for additional testing of stored specimens were included in this study; children aged 24-119 months were selected via simple random sampling and tested based on available specimens.

**Statistical analysis:** we estimated the overall and state-level seroprevalence, expressed as percentages with 95% CIs, against poliovirus types 1, 2, and 3 by age strata and by socio-demographic groups: sex, region, type of residence, water source, toilet type, and wealth index. The NAIIS 2018 wealth Index was constructed using household characteristics, asset ownership variables, services, and amenities that were present in both urban and rural surveyed areas, assumed to be good indicators of economic status [16]. The wealth index was presented as low, middle, or high income [13]. The distribution of poliovirus neutralization titers is presented using the median log2 and 95%CI for each of the three serotypes. Specimens without detectable neutralizing antibodies (<3.00 log2) are given a value of 2.50 log2. We conducted logistic regression analyses to assess factors associated with low seroprevalence including sex, age, region, toilet type, and water source. We documented the number of mOPV2 polio campaigns held during 2016-2018 [17] before NAIIS and compared it with the seroprevalence rates for children aged <24 months by state. All data analyses were performed in SAS 9.4 (SAS Institute Cary NC, USA).

**Ethical consideration:** the study proposal for testing of residual samples after NAISS HIV testing received approvals from the Nigeria Health Research and Ethics Committee (NHRECC) and the University of Maryland Baltimore Institutional Review Board. The US CDC approved the project as a non-research public health surveillance activity. The participant´s parents or guardians gave written informed consent and permission for residual samples to be stored and tested for diseases of public health importance.

## Results

In total, 1,185 samples were tested for types 1, 2, and 3 poliovirus-neutralizing antibodies, including 238 samples from children aged <24 months. Half (n=597, 50.4%) of the children were male, 49.4% were aged ≥ 60 months, and 50.8% were living in rural areas. The main water sources for participants were pipe-borne water (63%). Only 25% had access to a flush toilet system. Most of the children (61.6%) were in the low-wealth index category (Table 1).

**Table 1:** sociodemographic characteristics of children, Nigeria, 2018

Variable	Frequency (n=1185)	Percent
**Age (months)**		
0-11	90	7.6
12-23	148	12.5
24-35	135	11.4
36-59	227	19.2
60-119	585	49.4
**Sex**		
Female	588	49.6
Male	597	50.4
**State**		
Jigawa	306	25.8
Lagos	232	19.6
Sokoto	329	27.8
Yobe/Borno	318	26.8
**Region**		
South	232	19.6
North	953	80.4
**Water source**		
Piped water/bottle/bore hole	754	63.6
Well/spring water	368	31.1
Rainwater/tanker/cart water	50	4.2
Others	13	1.1
**Toilet type**		
Flushed system	295	24.9
Pit latrine	734	61.9
Composing/bucket/hanging latrine	6	0.5
No facility/bush	143	12.1
Others	7	0.6
**Wealth**		
Low	730	61.6
Middle	278	23.5
High	177	14.9
**Residence**		
Urban	583	49.2
Rural	602	50.8

Overall, the seroprevalence of poliovirus-neutralizing antibodies was 95.7% (95% CI: 94.7-96.7) for type 1,88.6% (95% CI: 86.8-90.4) for type 2, and 89.3% (95% CI: 87.4-91.3) for types 3. Seroprevalence for type 1 and type 3 polio antibodies was higher in the southern region than northern region. Among all the states in the northern region, Sokoto State had the lowest seroprevalence rates (Table 2). Seroprevalence was higher among those residing in urban areas compared to those residing in rural areas for all three polio types. By water source, those with piped water, bottle, or borehole had higher seroprevalence than those with well or spring water for all three types. By toilet type, those with a flushed system had slightly higher seroprevalence against poliovirus type 1 (97.5, 95% CI: 95.9-99.1) than those with pit latrine (94.4, 95% CI: 92.8-95.9) and those with no facility/bush (95.2, 95% CI: 92.9-97.5). Those children from high-wealth-index families had higher seroprevalence for all three types than those from low-wealth-index families.

**Table 2:** percent seroprevalence of poliovirus-neutralizing antibodies among children

Variable	Seroprevalence		
	Poliovirus type 1 (95%CI)	Poliovirus type 2 (95%CI)	Poliovirus type 3 (95%CI)
**Age (months)**			
0-11	82.9 (76.1-89.6)	57.1 (44.1-70.2)	69.9 (58.6-81.2)
12-23	88.0 (81.3-94.6)	54.6 (44.8-64.4)	81.8 (74.4-89.1)
24-35	94.7 (91.7-97.8)	78.2 (69.5-87.0)	86.3 (80.0-92.6)
36-59	96.2 (94.1-98.4)	93.7 (90.1-97.3)	89.3 (84.7-93.9)
60- 119	98.1 (97.1-99.1)	96.5 (94.8-98.1)	92.9 (90.5-95.4)
**State**			
Jigawa	93.8 (91.2-96.4)	86.6 (83.2-89.0)	86.4 (82.6-90.3)
Lagos	99.0 (97.7-100.0)	89.9 (86.3-93.4)	93.1 (89.2-97.0)
Sokoto	90.1 (86.9-93.2)	83.6 (79.7-87.5)	82.1 (77.7-86.5)
Yobo/Borno	95.9 (93.8-98.0)	91.6 (88.5-94.6)	90.7 (87.3-94.0)
**Place of residence**			
Urban	96.8 (95.6-98.0)	89.7 (87.3-92.0)	91.0 (88.3-93.6)
Rural	93.9 (92.0-95.7))	86.7 (84.0-89.4)	86.5 (83.5-89.5)
**Water source**			
Piped/bottled/borehole	97.2 (96.1-98.2)	89.4 (87.3-91.6)	91.4 (89.0-93.7)
Well/spring	90.4 (87.4-93.5)	85.0 (81.4-88.5)	81.6 (77.4-85.8)
Rainwater/tanker/cart	96.1 (91.8-100.0)	90.7 (82.9-98.6)	90.6 (83.7-97.5)
Others	100.0	94.0 (80.2-100.0)	100.0
**Toilet type**			
Flushed system	97.5 (95.9-99.1)	89.1 (85.6-92.5)	91.5 (87.7-95.3)
Pit latrine	94.4 (92.8-99.1)	88.6 (86.5-90.8)	87.7 (85.3-90.1)
No facility/bush	95.2 (92.9-97.5)	85.6 (80.1-91.1	88.3 (82.6-94.1)
**Wealth**			
Low	93.6 (91.8-95.3)	87.3 (85.0-89.5)	85.7 (83.0-88.3)
Middle	96.2 (94.1-98.3)	89.1 (85.1-93.1)	87.7 (82.5-92.9)
High	98.9 (97.9-100.0)	90.3 (86.5-94.1)	97.4 (95.1-99.6)

**Figure 1:**
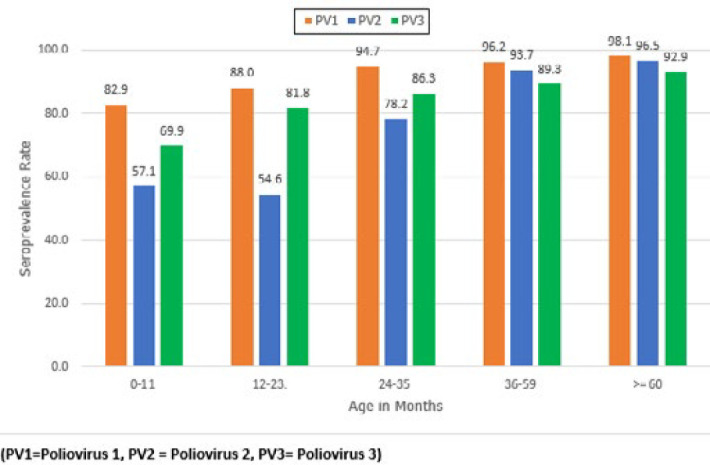
percent seroprevalence of poliovirus neutralizing antibodies for types 1, 2, 3; disaggregated by age group; Nigeria (Borno, Jigawa, Lagos, Sokoto, Yobe States), 2018

The seroprevalence generally increased with age (Figure 1). The age groups of 0-11 months and 12-23 months had the lowest seroprevalence for types 1, 2, and 3 compared to older age groups (Table 2). Children <24 months had the lowest PV2 seroprevalence rate of 55% (57% for children 0-11 months and 54% for 12-23 months of age (Table 2) irrespective of the number of mOPV2 campaigns conducted in the same year prior to NAIIS.

Median reciprocal titers of polio-neutralizing antibodies expressed on the log2 scale were 9.01 (95% CI, 8.74-9.27) for type 1,7.41 (95% CI, 7.21-7.62) for type 2, and 7.19 (95% CI, 6.97-7.40) for type 3 (Table 3). Median antibody titers for type 2 and 3 were higher among children >aged 24 months than those 0-23 months. Type 2 median antibody reciprocal titers were lowest in children 0-11 months (3.29, 95% CI: 2.65-3.93) and 12-23 months (3.18, 95% CI: 2.73-3.63). Type 3 median antibody titers were lowest in children 0-11 months (5.13, 95% CI: 3.55-6.71). Multivariable regression analysis showed that females (p=0.0184), aged 0-23 months (p<0.0001), from the northern region (p=0.0039) were associated with lower seroprevalence for poliovirus type 1. Females (p=0.0354) aged 0-23 months (p<0.0001) were associated with lower seroprevalence for poliovirus type 2. Male and female children aged 0-23 months (p<0.0001) and well water source (p=0.0288) were associated with lower seroprevalence for poliovirus type 3 (Table 4).

**Table 3:** distribution of poliovirus neutralizing antibody log2 titers among children by age group, Nigeria, 2018

Variable	Seroprevalence		
	Poliovirus type 1 neutralizing antibody titer (95%CI)	Poliovirus type 2 neutralizing antibody titer (95%CI)	Poliovirus type 3 neutralizing antibody titer (95%CI)
**Age (months)**			
0-11	6.62 (4.54-8.70)	3.29 (2.65-3.93)	5.13 (3.55-6.71)
12-23	8.33 (7.14-9.51)	3.18 (2.73-3.63)	7.79 (6.82-8.77)
24-35	9.98 (9.28-10.69)	6.72 (5.24-8.20)	7.91 (6.83-8.98)
36-59	9.39 (9.04-9.73)	8.51(7.89-9.14)	7.98 (7.51-8.46)
60- <120	8.47 (8.28-8.67)	7.75 (7.43-8.07)	6.65 (6.21-7.09)

**Table 4:** logistic multiple regression analysis of risk factors for low seroprevalence against poliovirus types 1, 2, 3, Nigeria, 2018

Factor	Seroprevalence if factor present, %	Seroprevalence if factor absent, %	Univariable regression, P-value	Multivariable regression, P-value
**Type 1**				
Sex female	94.4617	96.9221	0.0203	0.0184
Age 0-23 months	85.9992	97.2536	<.0001	<.0001
Live in Northern state	93.7119	99.0022	0.004	0.0039
Access to latrine (vs defecation in open)	97.4764	94.5869	0.0252	-
Water source (well/spring water vs rest)	90.4335	97.131	<.0001	-
**Type 2**				
Sex female	86.5628	90.5629	0.0494	0.0354
Age 0-23 months	55.5603	93.8434	<.0001	<.0001
Live in Northern state	87.8223	89.8603	0.3456	-
Access to latrine (vs defecation in open)	89.0791	88.2823	0.7054	-
Water source (well/spring water vs rest)	84.971	89.5673	0.0292	-
**Type 3**				
Sex female	89.1165	89.5596	0.8281	-
Age 0-23 months	77.2129	91.2695	<.0001	<.0001
Live in Northern state	87.0568	93.1058	0.0301	-
Access to latrine (vs defecation in open)	91.5045	87.9663	0.1524	-
Water source (well/spring water vs rest)	81.6356	91.4168	<.0001	0.0288

In 2018, all northern states had carried out two to four rounds of mOPV2 campaigns and one intensified IPV round 1-12 weeks prior to the NAIIS sample collection. However, seroprevalence for type 2 in children 0-23 months was low; no mOPV2 or intensified IPV campaigns were held in Lagos State (Table 5).

**Table 5:** number of PV2 campaigns using mOPV2 vaccines, between 2017-2018, prior to Nigeria National HIV/AIDS Indicator and Impact Surveysample collection in five Nigerian states

State	Dates samples collected in NAIIS	mOPV2 campaigns conducted in 2018	Intensified IPV campaign, 2018	Number of mOPV2 or fIPV campaigns 2017- 2018 and weeks prior to date of NAIIS sample collection	Seroprevalence poliovirus type 2 neutralizing antibody (95%CI) (0-23 months of age)
Borno and Yobe	November 26, to December 18, 2018	28 July- 1 August 1-4 sept, 6-9 Oct	3-13 November	2018: two mOPV2 rounds in Borno, four in Yobe, ~6-9 weeks before NAISS 2017: One mOPV2 round	68.4% (56.1% - 80.6%)
Jigawa	August 25, to September 9 2018	10-13 May; 26-29 May,	7-12 June	2018: four mOPV2 rounds ~1 - 9 weeks before NAIIS 2017: one mOPV2	50.8% (38.5% - 63.1%)
		1-4 sept, 6-9 Oct	3-13 November		
Sokoto	October 11, to October 24, 2018	10-13 May; 26-29 May,	15-20 July	2018: four mOPV2 rounds ~1-12 weeks before NAIIS 2017: two mOPV2 + 1 fIPV round (s)	54.5% (41.4% - 67.5%)
		1-4 sept, 6-9 Oct	3-13 November		
Lagos	July 19 to August 30, 2018	No mOPV2 campaigns	No IPV campaign	2018: no mOPV2 2017: no mOPV2 round	52.7% (34.7% - 70.7%)

PV2 = Polio virus type 2; NAIIS = Nigeria HIV/AIDS indicator and impact survey; fIPV = fractional doses of the inactivated poliovirus vaccine; mOPV2 = Monovalent type 2 oral polio vaccine

## Discussion

This study showed overall higher seroprevalence in children aged <10 years of age for poliovirus types 1 (96%) than types 2 and 3 (88.6% and 89%, respectively). However, the post-switch population of children <24 months of age had lower seroprevalence for all serotypes, especially type 2, at a seroprevalence rate of 55%. This age group is the population most vulnerable to poliomyelitis [18]. Regression analysis confirmed that with all other variables controlled, children <24 months had significantly lower type 2 immunity despite opportunities for IPV and mOPV2 vaccination. This finding is consistent with prior studies in Nigeria [9,15], but it is concerning due to the unprecedented number and geographic spread of cVPV2 outbreaks in Nigeria following the switch.

Prior to sample collection for NAIIS In 2018, at least 2 mOPV2 outbreak response SIAs were carried out in each of the included northern states, type 2 seroprevalence ranged 50-68%. Routine immunization coverage with IPV is low in the northern states, with the 2018 DHS survey results ranging from 20-45% in the northern states, compared to 87% in Lagos State [8]. Low IPV coverage means many children are vulnerable to paralysis if infected with cVPV2. There were 34 cVDPV2 AFP cases reported in the northern states during 2016-2018 compared with none in Lagos State during this period [17]. The indicator of the quality of the cVDPV2 outbreak response mOPV2 SIAs in these northern states was suboptimal; lot quality assurance sampling (LQAS) surveys [19] results at the local government area level ranged 65-85% passing at a 90% threshold. With low-quality SIAs, a substantial proportion of unimmunized children remains. Concordant to the LQAS survey results, type 2 seroprevalence in children aged <24 months in this study was only 55%.

There were several limitations to the study. First, it was underpowered. The number of samples tested and used in analyses was less than the estimated sample size since NAIIS sampling was not intended to assess poliovirus seroprevalence; in addition, some stored samples had insufficient serum volume for testing. Nonetheless, this study had one of the larger sample sizes conducted for seroprevalence studies in Nigeria [10,12,15]. Second, because of the small number of samples available from Borno and Yobe State, we combined Borno and Yobe as a cluster and there are no state-specific estimates. Third, data were not collected on the number of OPV or IPV vaccine doses that participating children received as part of the RI program or during SIAs. Thus, we could not compare seroprevalence survey data with the number of vaccines each child had received. However, we had data on mOPV2 SIAs and IPV intensification by state to indicate opportunities for type 2 seroconversion.

Despite these limitations, this study reflects low immunity in children born post-switch in the northern states which indicates low RI IPV coverage, and poor-quality mOPV2 and IPV SIAs that repeatedly did not reach target populations. As anticipated, our findings reflect that the switch from tOPV to bOPV, prior to having sufficient IPV coverage, contributed to type 2 immunity gaps. With the novel coronavirus disease 2019 (COVID-19) pandemic, when RI coverage decreased further and bOPV2 campaigns were not conducted, waning immunity against types 1 and 3 will occur as well unless bOPV and IPV administered in RI are improved in parallel with preventive bOPV SIAs reinstated and type 2 response SIAs are of higher quality. Our data indicate that a large proportion of children in the northern states were missed during mOPV2 outbreak response SIAs. Lagos State also had low type 2 immunity among post-switch children aged <24 months as no mOPV2 response SIAs were implemented during the study period.

Implementation or improvement of several strategies would help to interrupt cVDPV2 outbreaks and improve RI coverage of poliovirus vaccines. First, Nigeria could work on performing higher-quality OPV SIAs with a heightened focus on reaching populations that have been repeatedly missed during campaigns. Chronically missed children and settlements sustain cVDPV2 transmission. Therefore, careful evaluations of all settlements in the catchment area, frequent mapping using geospatial technology, strong pre-campaign preparedness and enhanced supervision of SIA teams are critical for improving campaign quality and reducing the number of repeatedly missed children [20].

Other approaches can be implemented like nomadic population enumeration, which helped identify several missed children in 2014, to ensure more children are reached with vaccines [21]. To prevent seeding new cVDPV2 outbreaks with suboptimal mOPV2 SIAs, novel OPV type 2 (nOPV2) can be used for type 2 outbreak response SIAs. The nOPV2 is modified to be more genetically stable than Sabin mOPV2 and less likely to be associated with the emergence of cVDPV2 in low immunity settings [22]. In clinical trials, nOPV2 provides comparable seroconversion against type 2 poliovirus [23]. Following emergency use authorization by the World Health Organization (WHO), nOPV2 is now being used in Nigeria [22,24] and other countries, although supply has been limited. Secondly, while improving outbreak response SIAs, there could be a parallel effort on increasing RI coverage. Improving political, social, and cultural interventions can ensure caregivers attend RI scheduled visits, as well as take children to receive poliovirus vaccines during SIAs. Thirdly, IPV intensification can be conducted in states with low polio serotype 2 coverage, which has been effective in other settings to improve immunity for all three serotypes [22,25,26]. Like OPV SIAs, which achieved higher LQAs results relative to the IPV campaigns in 2018 in Nigeria [17], similar preparedness steps can be taken to improve IPV coverage.

Since lack of access to potable water and being a girl were risk factors for lower type 2 polio seroprevalence, GPEI and local leaders could engage with ministries in charge of water and sanitation to enhance access over time. In the interim, attention could be paid to sex disparities wherein females may be more selectively missed during SIAs campaigns and have decreased access to RI services. Nigeria introduced a second dose of IPV in September 2021; the current vaccination schedule includes IPV at ages 6 weeks and 14 weeks. Every effort to get infants to receive both IPV doses can be put in place while also enhancing all efforts to ensure quality OPV campaigns.

**Implication of the study findings:** the findings show that type 2 immunity is low in the selected states in children aged 0-23 months even after several outbreak response campaigns with mOPV2. Increase focus on reaching children aged 0-23 months during outbreak responses with type 2 OPV campaigns, while improving routine immunization coverage with all poliovirus vaccines.

## Conclusion

This study showed high seroprevalence rates against PV types 1, 2, and 3 in children aged 24-119 months. However, children born after the switch (<24 months) had low immunity against PV2 despite two or more rounds of outbreak responses using mOPV2. Age, access to clean water sources and latrines were associated with lower polio seroprevalence indicating immunity gaps. Strategies to increase routine immunization coverage, especially for post-switch children including periodic intensification of routine immunization and high-quality polio campaigns can increase immunity against the polio virus.

**Disclaimer:** the findings and conclusions in this report are those of the authors and do not necessarily represent the official position of the Centers for Disease Control and Prevention.

### What is known about this topic

- Past serosurveys in Nigeria have shown variable seroprevalence rates for anti-polio antibodies for types 1, 2, and 3.

### What this study adds

- Post-tOPV switch and introduction of IPV, type 2 immunity is still low especially in children less than 2 years old;
- Being female, age < 24 months and well source of water are risk factors for low seroprevalence rates.
